# The management of severe eczema in pregnancy

**DOI:** 10.1016/j.clinme.2024.100282

**Published:** 2024-12-27

**Authors:** E Keeling, CH Smith, RT Woolf

**Affiliations:** a St John's Institute of Dermatology, Guy's and St Thomas' NHS Foundation Trust, London, United Kingdom; b King's College London, London, United Kingdom

**Keywords:** Eczema, Atopic dermatitis, Pregnancy, Skin directed treatments, Systemic treatment

## Abstract

Atopic eczema (eczema; also known as atopic dermatitis) is a chronic inflammatory skin condition. The burden of eczema can be very substantial with significant itch, skin pain, secondary infection, sleep disturbance and psychological distress. Eczema is common in pregnancy. It is therefore important to offer effective treatment to pregnant women, especially those with moderate to severe disease where the burden is greatest. When eczema cannot be adequately managed with skin-directed approaches such as topical preparations and/or phototherapy, systemic therapy may be required to achieve disease control and requires early input from dermatology specialists. The aim of this short review is to summarise this approach.

## Introduction

Atopic eczema (eczema; also known as atopic dermatitis) is a chronic inflammatory skin condition that is common, with an estimated cumulative lifetime prevalence of 10 % in the UK.^1^ Eczema can be associated with other atopic (allergic) conditions, including food allergy, asthma and hay fever.^2^ The burden of eczema can be very substantial with significant itch, skin pain, secondary infection, sleep disturbance and psychological distress.^3^

Eczema is common in pregnancy. It is therefore important to offer effective treatment to pregnant women, especially those with moderate to severe disease where the burden is greatest. For women planning conception, important issues in optimisation of eczema treatment include discontinuing any therapy with known teratogenic potential (specifically mycophenolate mofetil and methotrexate). Ongoing adjustment of therapeutic strategies throughout pregnancy is also key. When eczema cannot be adequately managed with skin-directed approaches such as topical preparations and/or phototherapy, systemic therapy may be required to achieve disease control and requires early input from dermatology specialists. The aim of this short review is to summarise this approach.

## Why is eczema common in pregnancy?

Eczema is a chronic inflammatory condition. Key processes contributing to its pathogenesis are: (1) disruption of the skin epithelial barrier, (2) immune dysregulation with upregulation of type 2 (allergic) immune responses and (3) alteration of the cutaneous microbiome, characterised by skin *Staphylococcus aureus* (*S aureus*) colonisation. There is also a strong genetic component, with genetic variation in genes encoding key components of these processes contributing to susceptibility to eczema.^4^

Eczema is common in women of childbearing age. It accounts for up to 50% of all dermatoses in pregnancy.^5^ Eczema activity during pregnancy is unpredictable. Previous studies have reported deterioration in eczema symptoms in 52%, 61% and 17% of pregnant individuals.^5^^,^^6^^,^^7^ Improvement in eczema during pregnancy is seen in 20% of women.^8^ In addition, many women develop new-onset eczema in pregnancy (atopic eruption of pregnancy), which morphologically and histologically resembles atopic eczema. However, atopic eruption of pregnancy is considered by some to be distinct to pregnancy as many women have no prior history of eczema, and the problem may resolve postpartum. Pregnancy induces immunological changes in the mother to allow the fetus to develop and to avoid rejection by the mother’s immune system. This includes a shift towards type 2 immune responses, which may predispose to the development of atopic eczema and atopic eruption of pregnancy. Other factors that may also contribute include hormonal changes in pregnancy (oestrogen and progesterone stimulate type 2 inflammation)^9^ and psychological/physical stress seen during pregnancy.^10^ From a management perspective, however, distinction between the two conditions is less important, since treatment strategies are the same.

## Making the diagnosis

The diagnosis of eczema is clinical. Typically, this comprises itchy (pruritic), red scaly patches at flexural sites, and/or ill-defined patches on the face, trunk or limbs. Skin type and ancestry can influence the clinical features. In women of African ancestry the patches are often papular, and may be extensor (rather than flexural) in distribution. Therefore palpation is also important. In skin of colour more generally, skin redness is often missed, so the extent and severity of the eczema are underestimated.^11^^,^^12^ A personal or family history of atopy is common. The clinical presentation of atopic eruption of pregnancy mimics that of atopic eczema. Differential diagnoses include other itchy conditions (Table 1) and other dermatoses of pregnancy (Table 2).

**Table 1:** Important diagnoses to consider when making the diagnosis of atopic eczema (or atopic eruption of pregnancy) during pregnancy.

Diagnosis	Typical presentation
Scabies (*Sarcoptes* infestation)	New-onset, pruritic rash, typically affecting hands (palms), wrists, feet, genital regions. Itchy rash in close contacts. Tip: look for burrows, papules and pustules in these anatomical sites.
Allergic/irritant contact eczema (dermatitis)	Due to contact with an external irritant or allergen a few hours or days after exposure. Can be any part of the exposed body, but commonly involves the hands and/or face. Morphology of the eczema patches is similar/identical to atopic eczema (and can occur concurrently with atopic eczema). Tip: ask about onset of rash, potential triggers eg fragrances/nickel exposure (jewellery), occupation, hobbies.
Psoriasis	Less pruritic, well-circumscribed, flat-topped plaques with silvery adherent scales; typically symmetrical on extensor aspects of elbows and knees and scalp. Tip: examine post-auricular region, common site for psoriasis.
Drug eruption	New-onset rash, typically occurring within 2 weeks of introduction of a new medication. Widespread maculopapular eruption. Tip: this rash often starts on the trunk and then spreads to the limbs.
Ringworm (*Tinea corporis*)	Annular (circular) red patches, with central clearing and peripheral scaling. Usually asymmetrical and localised. Tip: look between third/fourth/fifth toes for co-existing tinea pedis (athlete’s foot).
Pityriasis versicolor	Yeast infection of skin. Multiple round/oval patches that most commonly occur on the back, chest and upper arms. Colour can vary from pink, red, brown or white with fine scale. Usually asymptomatic.

**Table 2:** Other dermatoses of pregnancy.

Dermatosis of pregnancy	Clinical presentation	Investigations	Prognosis
Polymorphic eruption of pregnancy	Urticarial papules and plaques (similar to nettle rash), usually first appearing within striae distensae, with periumbilical sparing, during the latter portion of the third trimester or immediately postpartum.	This is a clinical diagnosis. A skin biopsy will distinguish this from pemphigoid gestationis when necessary.	Does not usually recur and confers no maternal or fetal risks
Pemphigoid gestationis	Rare. Autoimmune blistering condition in pregnancy. Itchy small vesicles and/or larger blisters that typically begin around the periumbilical region. Onset in late pregnancy or the immediate postpartum period.	Sub-epidermal bullae with eosinophils and linear C3 deposition along the basement membrane zone (BMZ) on skin biopsy. Serum can be sent for indirect immunofluorescence, which will demonstrate linear C3 staining along the dermo-epidermal junction	Increased risk of prematurity and small-for-gestational-age neonates. The condition commonly recurs in subsequent pregnancies. Early referral to dermatology is indicated.
Intrahepatic cholestasis of pregnancy	Intense generalised itch that often starts on the palms and soles, typically in the last trimester. There are no primary skin lesions or rash, but patients can present with widespread excoriations at sites that can be reached.	Total serum bile acid levels, cholic acid, chenodeoxycholic acid, bilirubin, transaminases, GGT, PT, PTT and INR.	Risk of meconium-stained amniotic fluid, preterm birth, neonatal ICU admission, intrauterine death.

## Management approach

### Assessment

Active eczema can be highly symptomatic, with a major impact on the individual affected. Acknowledging this, enquiring about aggravating environmental factors, health beliefs about their eczema, and current treatment strategies is crucial. Avoiding certain foods to manage food allergy or intolerances (real or perceived) can lead to restricted diets that are important to identify and address to ensure adequate nutrition. Some individuals will give a history of recurrent herpes simplex infection, and this can become disseminated (so called ‘eczema herpeticum’). It is important to be aware of this particularly if complicating the last trimester/perinatal period, to prevent transmission to the newborn.

Eczema typically varies in severity from week to week, and even day to day, so evaluating level of control over time, as well as the extent of disease activity at presentation, will determine the general treatment strategy. Localised eczema can usually be effectively managed with topical treatments (creams and/or ointments), prescribed and managed in primary care / non-specialist settings. ‘Moderate to severe’ disease includes those with more extensive skin involvement (for example, more than 10% of the body surface area involved), and/or where the eczema results in significant functional impairment and/or high levels of distress, such as severe hand and foot eczema, or head and neck eczema. Indicators of poorly controlled disease include disrupted sleep, persistent distressing itch, recurrent ‘flares’ requiring A&E or primary care attendances, need for repeated courses of antibiotics or large volumes of topical corticosteroids (for example >100 g/week). In general, clinicians risk undertreating eczema during pregnancy. Women with atopic eczema may also tend to underuse their usual treatments because of concern about harm to their baby and confusing advice. Collectively, this can result in poorly controlled eczema with significant impact on their physical, psychological or social wellbeing,^13^ as well as other complications such as eczema flares, systemic corticosteroids and secondary infections. Clear information on what treatments can be used safely during pregnancy is crucial.

## Skin-directed treatments

### Topical treatment

The first-line treatment for most eczema remains topical treatments, including (1) regular widespread application of emollients and (2) local application of topical immunosuppressants to areas of active eczema, including topical corticosteroids and topical calcineurin inhibitors.

A moisturiser (emollient), ideally with a high lipid content and fewer potential allergens, used at least daily will help maintain skin barrier function. Ointments can be more effective, but often patients prefer cream-based emollients. An adult with eczema affecting a large body surface area can use up to 500 g emollient per week. Tepid bathing, limited to once daily, with avoidance of basic soaps, is also advised.^13^

Topical corticosteroids and topical calcineurin inhibitors are safe to use in pregnancy, with limited systemic absorption in most patients.^14^^,^^15^ Consideration should be given to using an appropriate potency topical steroid for the anatomical site being treated. Caution should be taken when treating special sites including the skin folds and face, in particular the periocular region and eyelids. Table 3 summarises topical corticosteroids’ potency and use on different anatomical sites. The fingertip unit guidance is useful in guiding patients how much topical treatment to use.^16^ Topical corticosteroids and topical calcineurin inhibitors can be used daily to active areas of eczema. Once eczema resolves and itch improves, application can be tapered to twice weekly, which if required can continue in a ‘proactive approach’ to reduce the frequency of further flares.^17^

**Table 3:** Topical corticosteroid potency: suitability for varying anatomical locations.

Potency	Example of topical corticosteroids	Anatomical sites and treatment duration
Mild/moderate	Hydrocortisone 1% ointment / clobetasone butyrate 0.05% ointment	Face, neck – avoid eyelids. Use daily until eczema resolving, then taper. Avoid long-term daily use. 2.5 fingertip units to treat face and neck.
Moderate	Clobetasone butyrate 0.05% ointment / betamethasone valerate RD 0.025% ointment	Flexural sites. Use daily until eczema resolving, then taper. Avoid long-term daily use. 3 fingertip units to treat one whole arm.
Potent	Betamethasone valerate 0.1% ointment / mometasone furoate 0.1% ointment	Trunks, limbs and scalp. Use daily until eczema resolving, then taper. Avoid long-term daily use. 8 fingertip units to treat each: • whole leg and foot • chest and abdomen • back 3 fingertip units to treat scalp.
Super potent	Clobetasol propionate 0.05% ointment	Trunks, limbs and scalp, for severely affected areas. Use with caution. Limit daily use to shortest period required – avoid extending beyond 2 weeks. See fingertip units outlined above.

### Phototherapy

Narrowband ultraviolet B phototherapy (NB-UVB) is the most common phototherapy modality used for the treatment of eczema.^1^^3^ NB-UVB can be useful if avoiding systemic treatments; however, it requires frequent hospital attendance (usually twice weekly) for treatment and it can take up to 8 weeks for significant improvement in eczema to be seen. Phototherapy is also not suitable for patients with very widespread active eczema (erythroderma) which may flare as a result of treatment, or in those with significant photosensitivity. Phototherapy may also exacerbate pregnancy-induced hyperpigmentation, such as melasma.^13^

## Systemic approaches

Systemic treatment is indicated when moderate to severe eczema is inadequately controlled with topical therapy, and/or phototherapy is not suitable/practical. Aspects to consider and manage include gestation of the pregnancy and the likely projected duration of therapy to be required, impact on infant vaccination schedules, and breastfeeding.

• Oral corticosteroids are not advised for the ongoing management of eczema in general, given their side effect profile. In pregnancy specifically, reported increased risks of maternal hypertension, pre-eclampsia, premature rupture of membranes, infection, diabetes,^18^ and when used in the first trimester a small risk of oral clefts in the newborn, are all additional reasons to avoid them.^19^ In practice, short courses of oral prednisolone are often prescribed as a rescue treatment for severe acute flares,^20^ particularly in primary care, and are an indication for specialist referral for more proactive management since rapid relapse on stopping the oral steroid is common.
• Ciclosporin is a calcineurin inhibitor and a commonly used first-line systemic treatment strategy, as it is generally well tolerated and effective. Helpfully, there is a moderate amount of data on the use of ciclosporin during pregnancy, predominantly from post-transplantation registries. These data suggest that rates of miscarriages and major birth defects are comparable to those observed in the general population.^21^ As in the general population, monitoring for ciclosporin-induced hypertension and/or renal impairment is important throughout use, in close collaboration with the obstetric team.
• More recently, a number of highly effective National Institue for Health and Care Excellence (NICE)-approved targeted biologic immunomodulators have been introduced for atopic eczema, with consequent increasing experience of use during conception and pregnancy. Dupilumab was the first of these to be licensed, a fully human monoclonal antibody targeting interleukin-4 (IL-4) and IL-13 signalling, leading to at least a 75% improvement in baseline disease severity by 16 weeks.^22^ Animal studies do not indicate direct or indirect harmful effects with respect to reproductive toxicity. As with other therapeutic monoclonal antibodies, dupilumab crosses the placental barrier from week 20 of gestation, with the highest transfer in the third trimester. The summary of product characteristics (SmPC) advises that dupilumab can be used during pregnancy if the potential benefit justifies any (uncertain) risk to the fetus.^23^ There are limited high-quality data on the use of dupilumab in pregnancy; however, initial case reports / case series of dupilumab treatment in pregnancy indicate good eczema control and no harm.^24^^,^^25^^,^^26^ In practice, women with severe eczema who are well controlled on dupilumab pre-conception may choose to continue until completion of the first trimester, with ongoing use a matter to decide depending on historical disease severity and current status. Experience with the more recently introduced targeted biologic therapies (eg tralokinumab and lebrikizumab) is more limited, and the SmPC correspondingly more conservative.^27^^,^^28^
• It is recommended that immunisation with live vaccines should be delayed until 6 months of age for children born to mothers who received immunomodulating biologics (including monoclonal antibodies as discussed above). Rotavirus is a live vaccine included in the UK newborn vaccination schedule. Infants born to mothers who received non-biological immunosuppressive therapy such as steroids, ciclosporin or azathioprine at any time during their pregnancy can safely have the rotavirus vaccine at the appropriate age.^29^
• Treatments of limited benefit or to avoid. Antihistamines are not an effective treatment for eczema and are not advised as a primary or long-term treatment for eczema in pregnancy.^30^ In the last trimester, the sedative properties of some antihistamines (eg chlorphenamine) can also negatively impact on the baby. Azathioprine is compatible with pregnancy, but rarely used for eczema now that there are safer and more effective options available. Methotrexate, mycophenolate mofetil and JAK inhibitors are contraindicated in pregnancy due to risk of teratogenicity. Discontinuation of these medications is advised in advance of conception, with different washout periods for each.^31^(Table 4)

**Table 4:** Systemic agents used in eczema, which are contraindicated in pregnancy.

Methotrexate	Effective contraception must be used during treatment with methotrexate and at least 6 months thereafter.
Mycophenolate mofetil	Women of childbearing potential must use at least one form of reliable contraception before starting therapy, during therapy, and for 6 weeks after stopping the therapy.
Upadacitinb	Women of childbearing potential have to use effective contraception during treatment and for 4 weeks following the final dose of upadacitinib.
Abrocitinib	Women of reproductive potential should be advised to use effective contraception during treatment and for 1 month following the final dose of abrocitinib.
Baricitinib	Women of childbearing potential have to use effective contraception during treatment and for at least 1 week after last dose of baricitinib.
• Impact of systemic therapies on infant vaccination schedules and breastfeeding. Therapeutic monoclonal antibodies cross the placenta from 16 weeks onwards. Given their long half-life, infants born to mothers on these therapies in their second or third trimester should not receive live vaccines until aged 6 months.^32^ Careful consideration should be given to safety of systemic agents in breastfeeding. A recent review provided updated guidance on this topic.^33^

## Complications of eczema

### Infection

Active eczema is colonised by *S aureus* and bacterial infection can lead to flares. Infected eczema should be treated with appropriate antiseptics and antibiotics, based on sensitivities and safety for use in pregnancy. Antiseptics used the UK include dilute bleach baths. The National Eczema Association provides guidance on their preparation.^34^ The decision to use systemic antibiotics should be guided by the severity of the infection. Maternal antibiotic exposure may be associated with increased risk of eczema in the child, and so systemic antibiotics should be rationalised.^35^ Herpes simplex virus can also infect underlying eczema, presenting as grouped vesicles, progressing to punched-out erosions, which can rapidly disseminate causing eczema herpeticum. This may represent a dermatological emergency and requires prompt diagnosis and treatment. Herpetic keratoconjunctivitis and meningoencephalitis are possible sequelae. Confirmatory viral swabs should be taken and systemic antiviral treatment started without delay.

### Psychological complications

Eczema can be associated with significant psychosocial morbidity. Chronic itch, associated sleep deprivation, and the burden of treatments are commonly reported issues. During pregnancy and in the postpartum period, additional concerns regarding the impact of eczema and its treatments on the newborn alongside other new stressors can have a detrimental impact on the mother’s mental health and wellbeing.

## Conclusion

Maternal eczema can be effectively managed with topical and systemic treatments in pregnancy. Untreated eczema is associated with other medical issues and a significant burden for the mother. Eczema in pregnancy is often undertreated and barriers to providing treatment include lack of knowledge and confidence in providing treatment to pregnant women.^13^ In cases of moderate-to-severe eczema that is failing to respond to topical treatment(s), early consultation with a dermatologist is important to support potential management with an appropriate systemic medication. Shared decision making with the expectant mother is also vital; focusing on the burden/risk of untreated eczema, appropriate treatment options and their effectiveness, treatment side effect profiles, safety data (often limited) in pregnancy and potential consequences of treatment avoidance. Helpful resources include medicine SmPCs, guidelines including NICE guidelines,^36^ American Academy of Dermatology guidelines,^37^ European guidelines,^38^ and the BUMPS (Best Use of Medicines in Pregnancy) website (UK) (patient-facing drug information available).^39^

## Key points

1) Eczema is common in the general population. Immunological changes in pregnancy can lead to increased activity in those with known eczema, or provoke new-onset eczema in those with no prior history.
2) Joint decision making with the mother is vital when considering potential treatment pathways. Untreated eczema increases the risk of secondary infection and has a profoundly negative impact on the affected individual’s quality of life.
3) The cornerstone of managing mild eczema remains topical treatments, including emollients, soap substitutes and topical corticosteroids.
4) Systemic treatment, where required, should be prescribed by a dermatologist, in conjunction with the patient’s obstetrician, with ciclosporin or dupilumab generally now considered first-line systemic agents.

## CRediT authorship contribution statement

**E Keeling:** Writing – original draft, Investigation, Formal analysis, Data curation. **CH Smith:** Writing – review & editing, Supervision, Formal analysis, Data curation, Conceptualization. **RT Woolf:** Writing – review & editing, Supervision, Formal analysis, Data curation, Conceptualization.

## Declaration of competing interest

The authors declare the following financial interests/personal relationships which may be considered as potential competing interests: Catherine Smith reports a relationship with biomap (biomap-IMI.eu) that includes funding grants. Catherine Smith reports a relationship with hippocrates (hippocrates-IMI.eu) that includes funding grants. Catherine Smith reports a relationship with psort (psort.org.uk) that includes funding grants. Catherine Smith reports a relationship with GSK that includes funding grants. Catherine Smith reports a relationship with Novartis that includes funding grants. Catherine Smith reports a relationship with Pfizer that includes funding grants. Catherine Smith reports a relationship with Abbvie that includes funding grants. Catherine Smith reports a relationship with Janssen that includes funding grants. Catherine Smith reports a relationship with Regeneron that includes funding grants. Catherine Smith reports a relationship with Boehringer Ingelheim that includes funding grants. Richard Woolf reports a relationship with Cleveland Clinic London Ltd that includes employment. Richard Woolf reports a relationship with Abbvie that includes consulting or advisory, funding grants, speaking and lecture fees, and travel reimbursement. Richard Woolf reports a relationship with Eli Lilly that includes funding grants, speaking and lecture fees, and travel reimbursement. Richard Woolf reports a relationship with LEO Pharma that includes consulting or advisory, speaking and lecture fees, and travel reimbursement. Richard Woolf reports a relationship with Janssen that includes speaking and lecture fees and travel reimbursement. Richard Woolf reports a relationship with Novartis that includes funding grants and speaking and lecture fees. Richard Woolf reports a relationship with Sanofi that includes speaking and lecture fees and travel reimbursement. Richard Woolf reports a relationship with UCB Inc that includes speaking and lecture fees and travel reimbursement. Richard Woolf reports a relationship with Amgen that includes funding grants. Richard Woolf reports a relationship with Aristea Therapeutics Inc that includes funding grants. Richard Woolf reports a relationship with Boehringer Ingelheim that includes consulting or advisory and funding grants. Richard Woolf reports a relationship with Bristol-Myers Squibb that includes funding grants. Richard Woolf reports a relationship with Celgene that includes funding grants. Richard Woolf reports a relationship with Galderma that includes funding grants. Richard Woolf reports a relationship with Pfizer that includes funding grants. If there are other authors, they declare that they have no known competing financial interests or personal relationships that could have appeared to influence the work reported in this paper.
